# Differential gene expression for carotenoid biosynthesis in a green alga *Ulva prolifera* based on transcriptome analysis

**DOI:** 10.1186/s12864-018-5337-y

**Published:** 2018-12-13

**Authors:** Yuan He, Yafeng Ma, Yu Du, Songdong Shen

**Affiliations:** Department of Cell Biology, College of Biology and Basic Medical Sciences, Soochow University, No. 199 Renai Road, SIP, Suzhou, 215123 China

**Keywords:** Green algae, Temperature response, qRT-PCR, Biological pathway, Gene ontology

## Abstract

**Background:**

Carotenoids are widely distributed in plants and algae, and their biosynthesis has attracted widespread interest. Carotenoid-related research has mostly focused on model species, and there is a lack of data on the carotenoid biosynthetic pathway in *U. prolifera* that is the main species leading to green tide, a harmful plague of floating green algae.

**Results:**

The carotenoid content of *U. prolifera* samples, that is the main species leading to green tide, a harmful plague of floating green algae at different temperatures revealed that its terpenoid was highest in the samples subjected to high temperature at 28 °C (H), followed by the samples subjected to low temperature at 12 °C (L). Its terpenoid was lowest in the samples subjected to medium temperature at 20 °C (M). We conducted transcriptome sequencing (148.5 million raw reads and 49,676 unigenes in total) of samples that were subjected to different temperatures to study the carotenoid biosynthesis of *U. prolifera*. There were 1125–3164 significant differentially expressed genes between L, M and H incubation temperatures, of which 11–672 genes were upregulated and 453–3102 genes were downregulated. A total of 3164 genes were significantly differentially expressed between H and M, of which 62 genes were upregulated and 3102 genes were downregulated. A total of 2669 significant differentially expressed genes were observed between L and H, of which 11 genes were upregulated and 2658 genes were downregulated. A total of 13 genes were identified to be involved in carotenoid biosynthesis in *U. prolifera*, and the expression levels of the majority were highest at H and lowest at M of incubation temperature. Both the carotenoid concentrations and the expression of the analysed genes were lowest in the normal temperature group, while low temperature and high temperature seemed to activate the biosynthesis of carotenoids in *U. prolifera*.

**Conclusions:**

In this study, transcriptome sequencing provided critical information for understanding the accumulation of carotenoids and will serve as an important reference for the study of other metabolic pathways in *U. prolifera*.

**Electronic supplementary material:**

The online version of this article (10.1186/s12864-018-5337-y) contains supplementary material, which is available to authorized users.

## Background

**Fig. 1:**
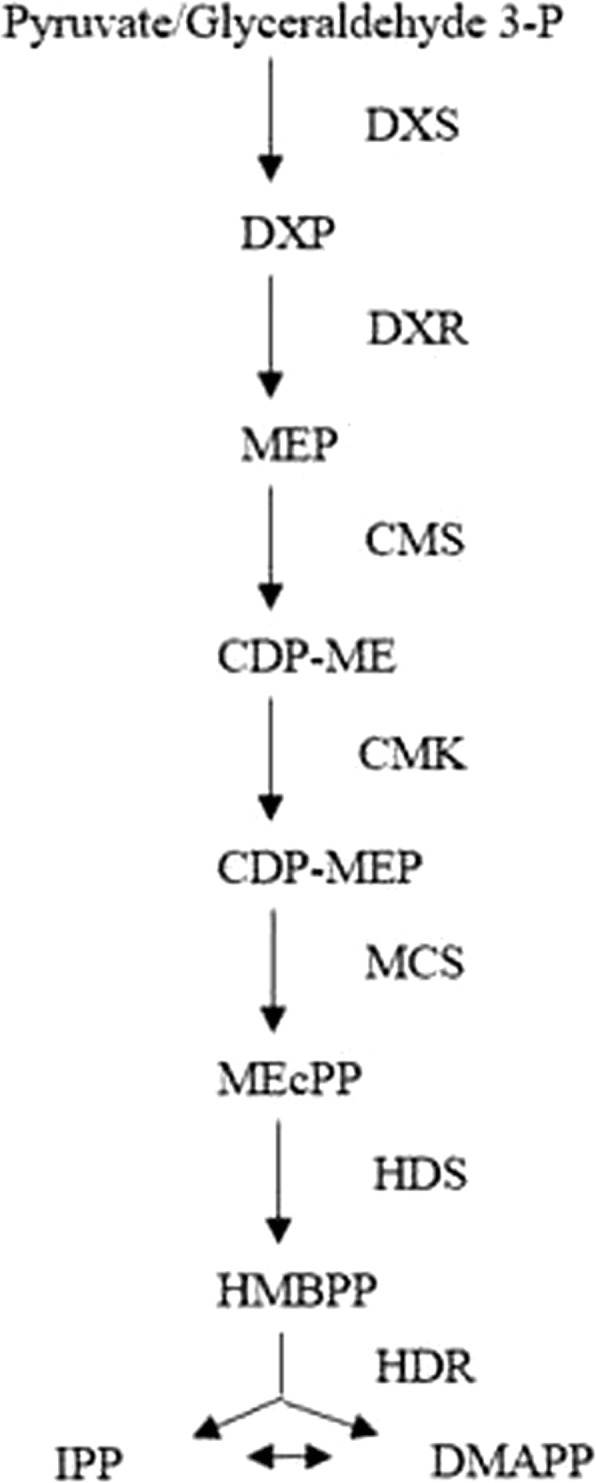
The MEP pathway in plants and algae. Seven enzymes participate in the steps of the MEP pathway. DXS: 1-deoxy-D-xylulose 5-phosphate synthase; DXR: 1-deoxy-D-xylulose 5-phosphate reductoisomerase; CMS: 2-C-methyl-D-erythritol 4-phosphate cytidyltransferase; CMK: 4-(cytidine-5-diphospho)-2-C-methyl-D-erythritol kinase; MCS: 2-C-methyl-D-erythritol 2,4-cyclodiphosphate synthase; HDS: 1-hydroxy-2-methyl-2-(*E*)-butenyl-4-diphosphate synthase; HDR: 1-hydroxy-2-methyl-2-(*E*)-butenyl-4-diphosphate reductase

Terpenoids are the most widely distributed natural compounds in nature, playing an important role in growth, development, light absorption, hormone synthesis, photoprotection and stress resistance in plants [1]. Terpenoids are the largest and most diverse volatiles released by plants [2]. Isopentenyl diphosphate (IPP) and dimethylallyl diphosphate (DMAPP) are the precursors for terpenoid synthesis. Two pathways participate in the biosynthesis of IPP and DMAPP in living organisms: the MVA pathway and the MEP pathway. In recent years, terpenoid metabolism in algae has been intensively studied, and there is adequate evidence that in green algae, the MVA pathway has been abandoned, and only the MEP pathway supplies the precursors of all cellular terpenoids [3]. The MEP pathway is not dependent on mevalonic acid (MVA) for IPP and DMAPP synthesis. There are seven enzymes involved in the pathway, including DXS, DXR, CMS, CMK, MCS, HDS and HDR (Fig. 1) [4]. These enzymes play an important role in regulating the pathway.

**Fig. 2:**
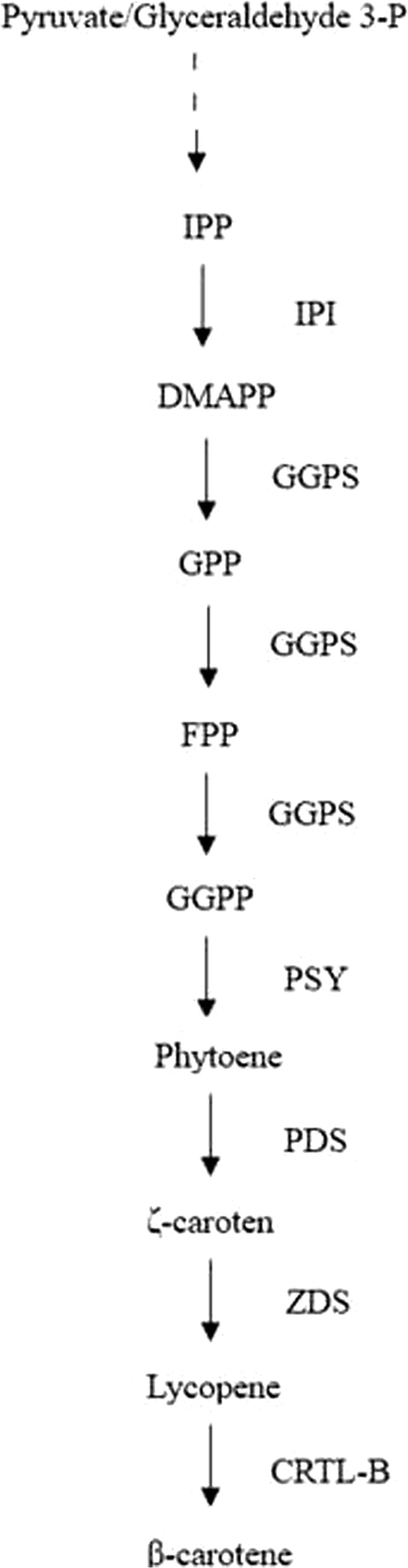
Carotenoid biosynthetic pathway in plants and algae. Six enzymes participate in the steps of carotenoid biosynthesis. IPI: isopentenyl diphosphate isomerase; GGPS: geranylgeranyl diphosphate synthase; PSY: phytoene synthase; PDS: phytoene desaturase; ZDS: ζ-carotene desaturase; CRTL-B: lycopene b-cyclase

Carotenoids are mostly C40 compounds consisting of eight isopentenyl-pyrophosphate units and comprise a class of natural pigments that belong to the tetraterpenoid group [5]. Carotenoids are generally synthesized through the terpenoid biosynthetic pathway in plant plastids. There are six enzymes involved in the pathway, including IPI, GGPS, PSY, PDS, ZDS and CRTL-B (Fig. 2) [6]. The formation of monoterpenes, sesquiterpenes, and diterpenes is activated by terpene synthases (TPS) in plants [7], however, scientists have found almost no TPS in algae [8], so it is critical to study the genes of the MEP pathway and the downstream pathway to understand carotenoid biosynthesis in algae.

*Ulva prolifera* (Ulvaceae, Chlorophyta), that is the main species leading to green tide, a harmful plague of floating green algae [9]. The macroalgae usually live in intertidal zones and have complex life histories and multiple reproductive events [10]. These features have been considered critical strategies to grow rapidly. Since 2007, *U. prolifera* blooms have occurred in the southern Yellow Sea continuously and led to a green tide disaster along the coast of the Shandong and Jiangsu provinces of China. This phenomenon had negative impacts on the local economy and environment [11–13]. Therefore, the study of *U. prolifera* is an increasing focus of scientists and has drawn considerable attention from the Chinese government [14, 15].

With the development of sequencing technology, transcriptome sequencing has been widely used in the study of various species. Transcriptomics revealed dynamics of photopigment [16], flavonoid [17] or terpenoid [18] synthesis in algae or land plants. Stress response of algae or cyanobacteria to desiccation [19], deprivation of essential element [20] or temperature [21] was portrayed by this technique. In green algae, transcriptome sequencing has been widely applied to study various biological processes [22–24].

Temperature is a key factor for terpenoid biosynthesis in algae [25]. In this study, we aimed to better understand the expression of key genes related to carotenoid biosynthesis in *U. prolifera* under different temperatures. All of the genes involved in the MEP pathway and the downstream pathway were screened, and their expression patterns were analysed through transcription data. Our results not only provide insight into carotenoid biosynthesis in *U. prolifera* but also provide an important reference for further study in *U. prolifera*. Terpenoids metabolism is an important metabolism of *U. prolifera*, we made a significative preliminary work for exploring the reason of the rapid formation of green tide in terms of the metabolic activities in the future.

## Results

### The carotenoid, chlorophyll (Chl) a and Chl b content of the L, M and H samples

**Fig. 3:**
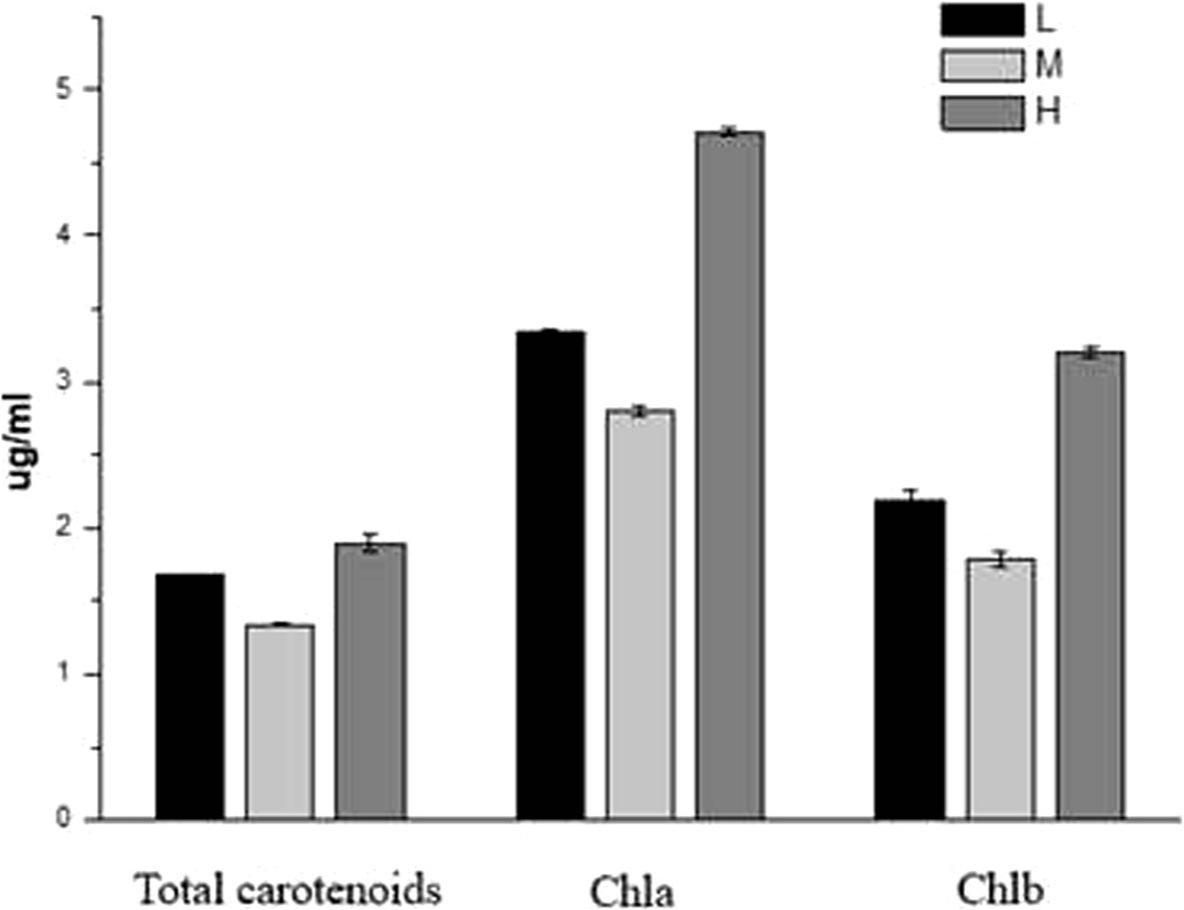
The concentrations of and total carotenoids, Chl a and Chl b in samples subjected to different temperatures. The absorbances at wavelengths of 470 nm, 646.8 nm and 663.2 nm were measured using a spectrophotometer, and the results were obtained using formulas [62]

The quantitative determination of carotenoid, Chl a and Chl b content was performed using a spectrophotometer. The concentrations of carotenoids, Chl a and Chl b in the L group were 3.34 ± 0.03 μg/ml, 2.20 ± 0.07 μg/ml and 1.67 ± 0.01 μg/ml, respectively. The concentrations of carotenoids, Chl a and Chl b in the M group were 2.80 ± 0.03 μg/ml, 1.78 ± 0.05 μg/ml and 1.34 ± 0.01 μg/ml, respectively. The concentrations of carotenoids, Chl a and Chl b in the H group were 4.72 ± 0.03 μg/ml, 3.2 ± 0.04 μg/ml and 1.90 ± 0.06 μg/ml, respectively (Fig. 3). The concentrations of terpenoids were highest in the samples that were subjected to high temperature, followed by the samples that were subjected to low temperature; the samples that were subjected to the medium temperature had the lowest terpenoid concentrations.

### Illumina HiSeq mRNA sequencing and transcriptomic assembly

A total of 148.5 million raw reads were generated from three libraries. After filtering out low quality sequences, approximately 145.6 million clean reads with 55.44% GC content were obtained, and high-quality (Q > 30) reads accounted for 96.02–96.14% of the reads (Table 1). We joined the reads into longer sequences that were continually extended into transcripts, and the longest sequence was selected as a unigene. A total of 49,676 unigenes with an N50 length of 1685 bp were obtained using the Trinity method. The total length was 62,289,934 bp, and the average length was 1253 bp. Among the unigenes, 39,942 unigenes (80.4%) were longer than 500 bp and 22,793 unigenes (45.9%) were longer than 1000 bp, and the results showed that the quality of the transcriptome data was sufficient for the subsequent analysis.

**Table 1:** Summary of RNA-seq data from three libraries

Sample	Raw reads	Clean reads	Valid bases	Q30	GC
L	49,527,516	48,539,744	94.19%	96.14%	52.61%
M	49,298,060	48,312,112	94.15%	96.02%	56.51%
H	49,717,316	48,755,416	94.33%	96.10%	57.21%

### Functional annotation of the transcriptome

The 49,676 unigenes were annotated using a variety of databases (Nr, KOG, GO, Swiss-Prot, eggnog, KEGG and Pfam). There were matches for 23,625 unigenes (47.56%) in the Nr database, 18,577 unigenes (37.40%) in the Swiss-Prot database, 12,652 unigenes (25.47%) in the KEGG database, 16,046 unigenes (32.30%) in the KOG database, 20,005 unigenes (40.27%) in the eggNOG database, 18,027 unigenes (36.29%) in the GO database, and 38 unigenes (0.08%) in the Pfam database.

**Fig. 4:**
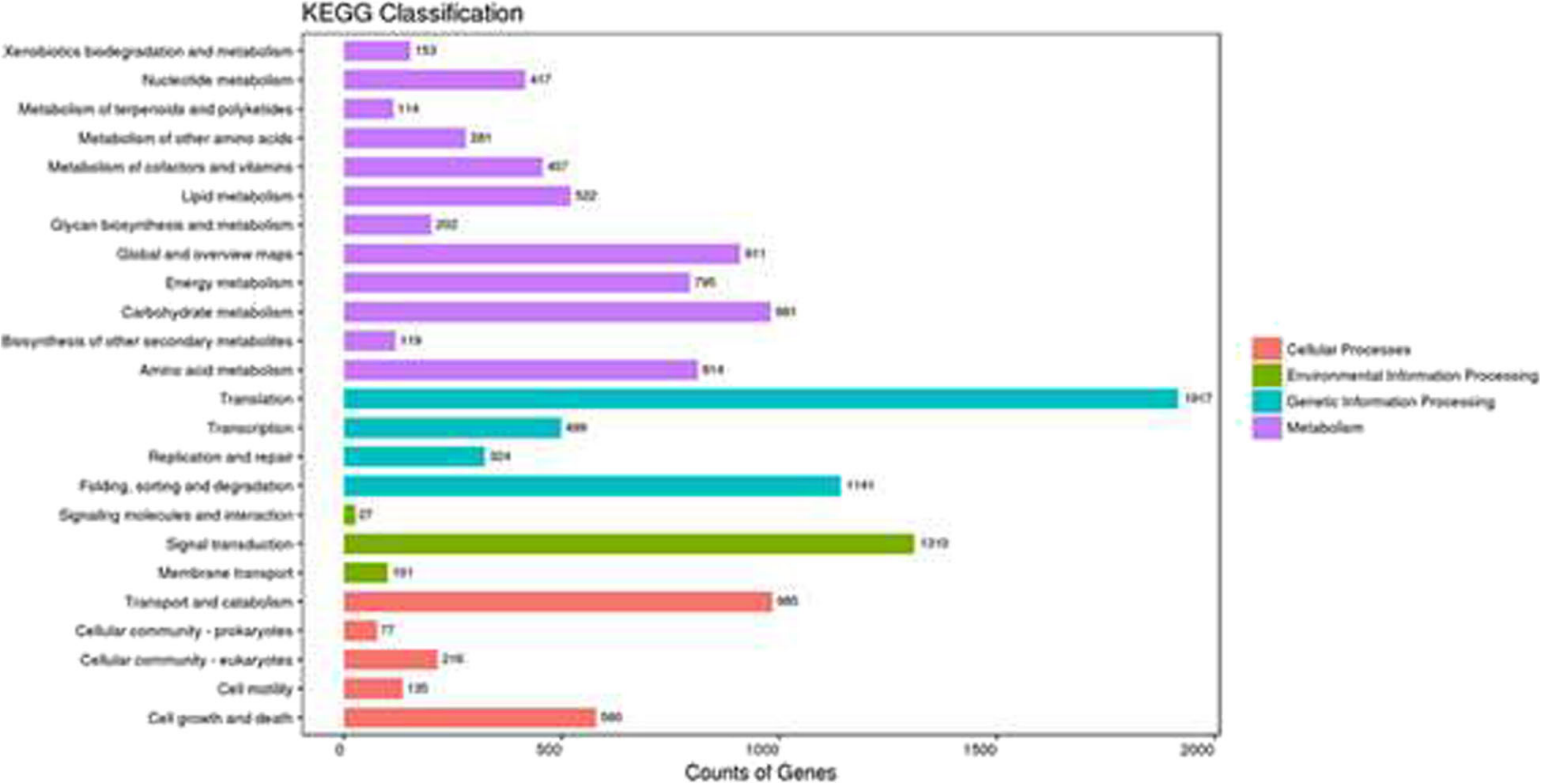
KEGG annotation of the non-redundant sequences of all the samples. The y-axis indicates the name of the KEGG metabolic pathway. The x-axis indicates the number of genes. The unigenes were divided into four branches according to the KEGG metabolic pathway: Cellular Processes, Environmental Information Processing, Genetic Information Processing, and Metabolism

A total of 12,652 unigenes were annotated in the KEGG database and were involved in 24 metabolic pathways (Fig. 4). The pathway with the most unigenes was ‘translation’ (1917 unigenes), followed by ‘signal transduction’ (1310 unigenes) and ‘folding, sorting and degradation’ (1141 unigenes). There were 144 unigenes that were predicted to participate in ‘metabolism of terpenoids and polyketides’, including ‘tetracycline biosynthesis’ (ko00253, 6), ‘geraniol degradation’ (ko00281, 10), ‘polyketide sugar unit biosynthesis’ (ko00523, 3), ‘terpenoid backbone biosynthesis’ (ko00900, 56), ‘limonene and pinene degradation’ (ko00903, 9), ‘carotenoid biosynthesis’ (ko00906, 22), ‘zeatin biosynthesis’ (ko00908, 3), ‘sesquiterpenoid and triterpenoid biosynthesis’ (ko00909, 4) and ‘biosynthesis of ansamycins’ (ko01051, 6).

GO functional annotations consist of three ontologies: cellular component, molecular function, and biological process. A total of 18,027 annotated unigenes were categorized into three ontologies with 56 GO terms (Additional file 1: Figure S1). The ‘cellular component’ category had the most unigenes (16183), followed by ‘molecular function’ (15,886 unigenes) and ‘biological process’ (15,205 unigenes). For the cellular component category, ‘cell’ and ‘cell part’ dominated in the ontology; ‘binding’ and ‘catalytic activity’ were the two most abundant terms in the ‘molecular function’ category, and the most highly represented terms in the ‘biological process’ category were ‘cellular process’ and ‘metabolic process’.

### Differentially expressed genes (DEGs) among the L, M and H samples

**Fig. 5:**
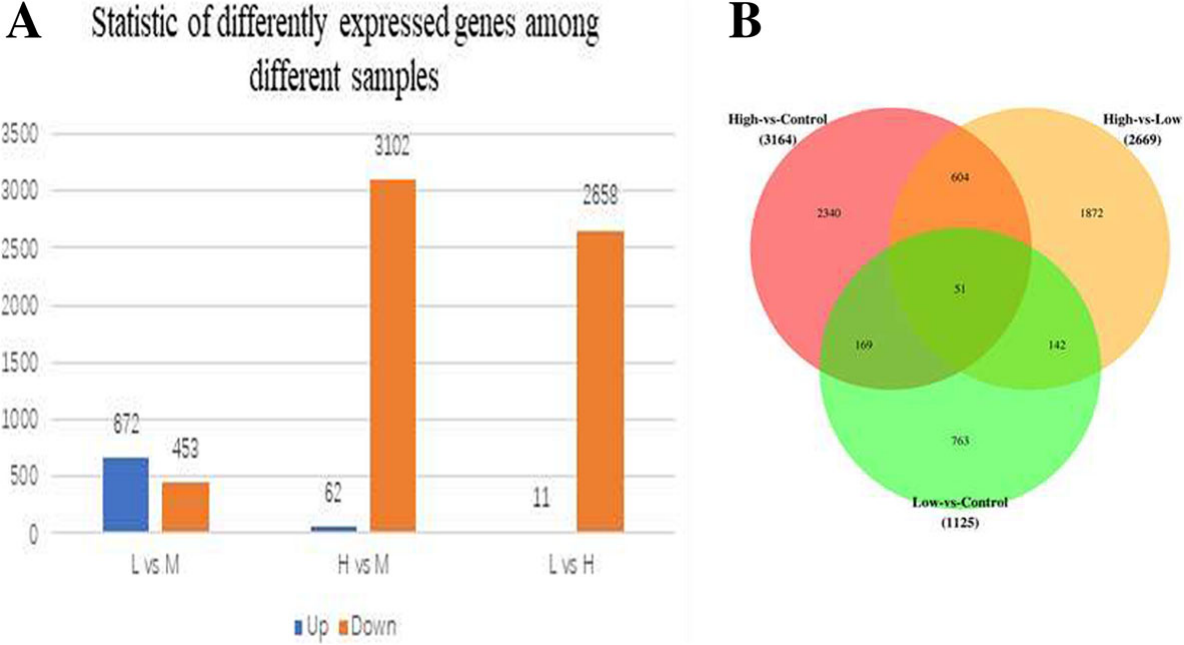
Differential expression analysis was performed for the L, M and H samples. **a** Number of differentially expressed genes (DEGs) among L, M and H. **b** Venn diagram of the DEGs among L, M and H

Differential expression analysis was performed for the L, M and H samples (Fig. 5). The results showed that there were 1125 significant differentially expressed genes between L and M, of which 672 genes were upregulated and 453 genes were downregulated in L compared to M. There were 3164 significant differentially expressed genes between H and M, of which 62 genes were upregulated and 3102 genes were downregulated in H compared to M. There were 2669 significant differentially expressed genes between L and H, of which 11 genes were upregulated and 2658 genes were downregulated L compared to H (Fig. 5a).

Venn diagrams of the differentially expressed unigenes among the L, M and H samples showed that 220 genes were significantly differentially expressed both between L and M and between H and M. These genes may play a key role in responses to temperature stress in *U. prolifera*. The number of genes that were differentially expressed between L and M only was 763; these genes may play a key role in responses to low-temperature stress in *U. prolifera*. The number of genes that were differentially expressed between H and M only was 2340; these genes may play a key role in responses to high-temperature stress in *U. prolifera* (Fig. 5b).

**Fig. 6:**
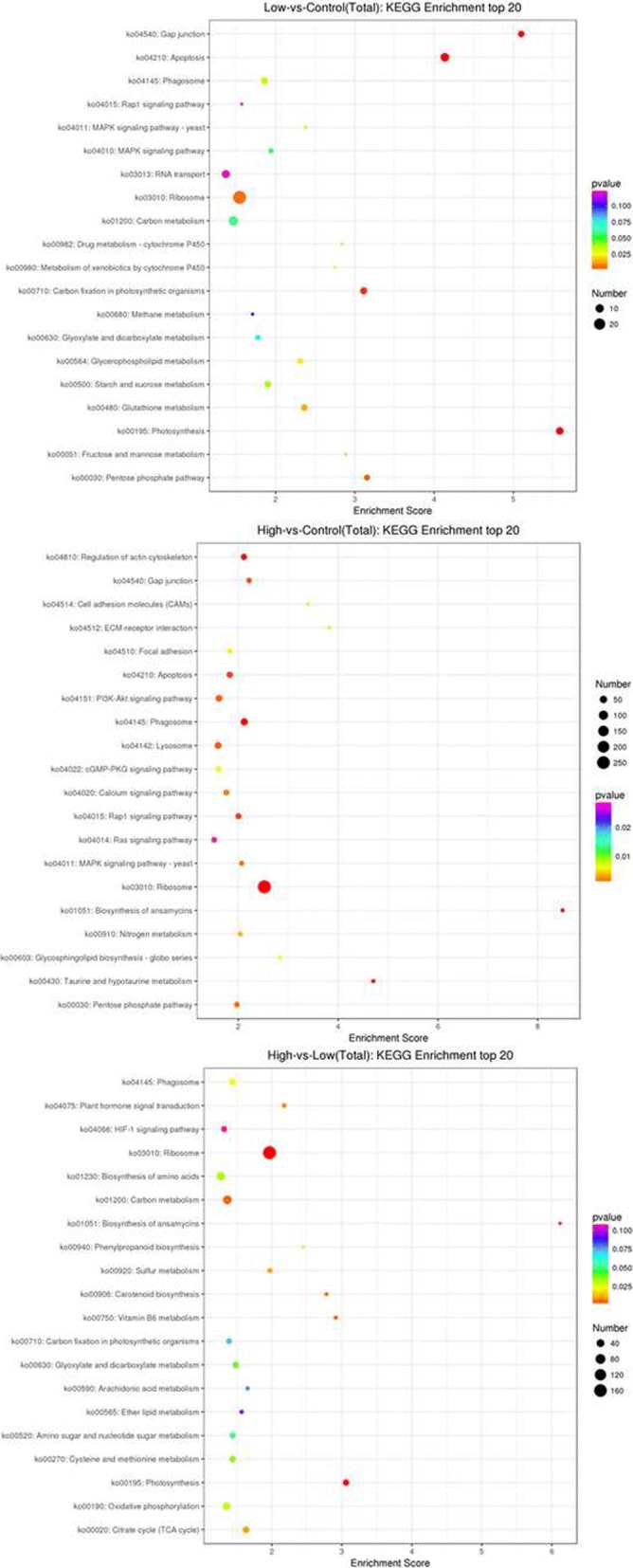
KEGG pathways significantly enriched in DEGs in comparisons of L, M and H

KEGG analysis was performed to further explore the functional classification and pathway assignment of the DEGs in the L vs M, H vs M and L vs H comparisons. Many genes were enriched for the gap junction, apoptosis, carbon fixation in photosynthetic organisms, photosynthesis, pentose phosphate pathway, carotenoid biosynthesis, linoleic acid metabolism, carbon metabolism, carotenoid biosynthesis, plant hormone signal transduction, and citrate cycle terms (Fig. 6). We used GO assignments to classify the functions of DEGs in pairwise comparisons of the cDNA libraries of samples subjected to different temperatures. The GO enrichment revealed 10 biological process categories, 10 cellular component categories and 10 molecular function categories that were enriched for the L vs M, H vs M and L vs H comparisons (Additional file 2: Figure S2).

### Genes involved in carotenoid biosynthesis

**Fig. 7:**
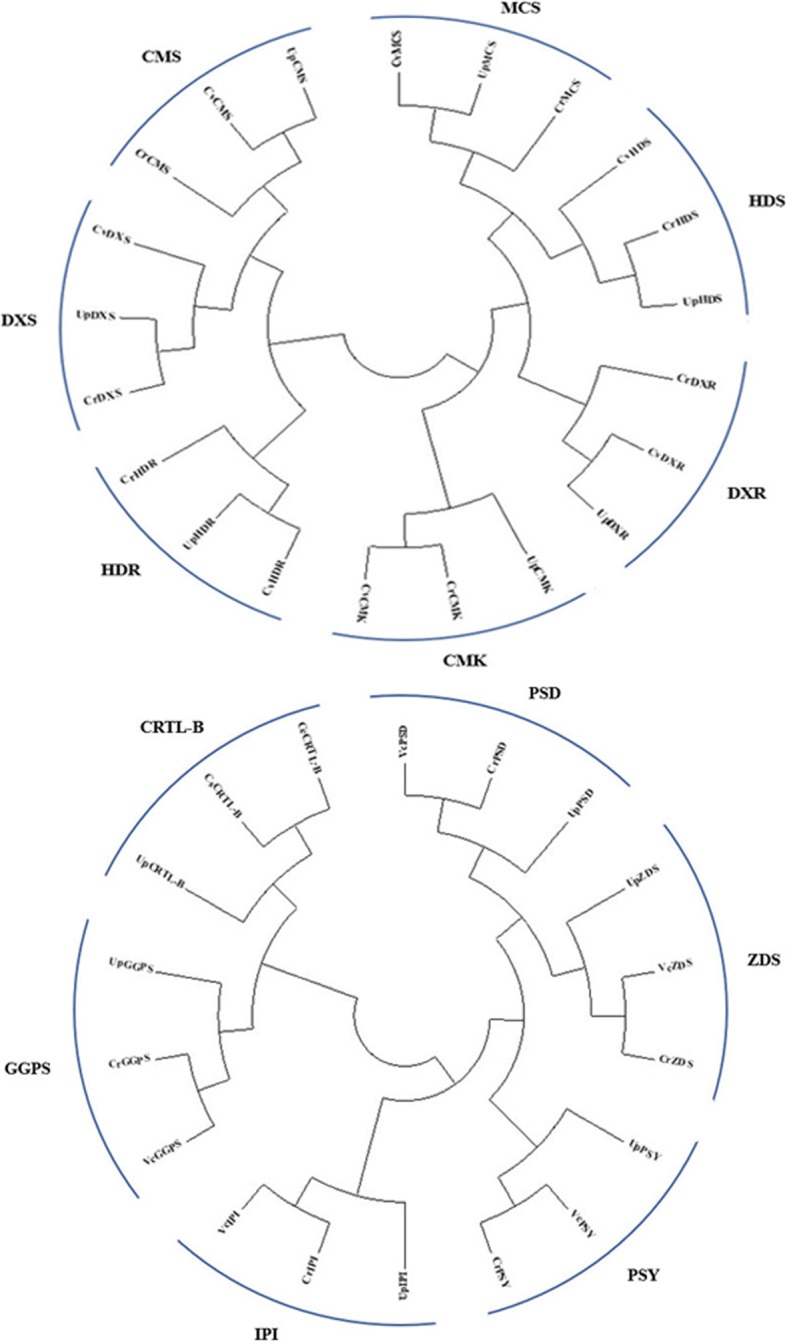
**a** Phylogenetic relationships of genes in the MEP pathway, including genes from *Chlamydomonas reinhardtii* and *Chlorella variabilis*. The genes were divided into 7 types: DXS, DXR, CMS, CMK, MCS, HDS and HDR. **b** Phylogenetic relationships of genes in the carotenoid biosynthetic pathway, including genes from *Chlamydomonas reinhardtii*, *Chlamydomonas eustigma*, *Chlorella sorokiniana*, *Chlorella variabilis* and *Volvox carteri*. The genes were classified as IPI, GGPS, PSY, PDS, ZDS and CRTL-B

All carotenoid biosynthetic pathway genes were identified in *U. prolifera* by analysing 56 unigenes that participate in terpenoid backbone biosynthesis and 22 unigenes that participate in carotenoid biosynthesis. The carotenoid biosynthesis genes in *Chlamydomonas reinhardtii*, *Chlamydomonas eustigma*, *Chlorella sorokiniana*, *Chlorella variabilis* and *Volvox carteri* were subjected to BLAST analysis with the annotated carotenoid biosynthesis genes in *U. prolifera*. The genes of the MEP pathway were divided into 7 types: DXS, DXR, CMS, CMK, MCS, HDS, and HDR (Fig. 7a); the genes of the carotenoid biosynthetic pathway were divided into 6 types: IPI, GGPS, PSY, PDS, ZDS and CRTL-B (Fig. 7b).

**Fig. 8:**
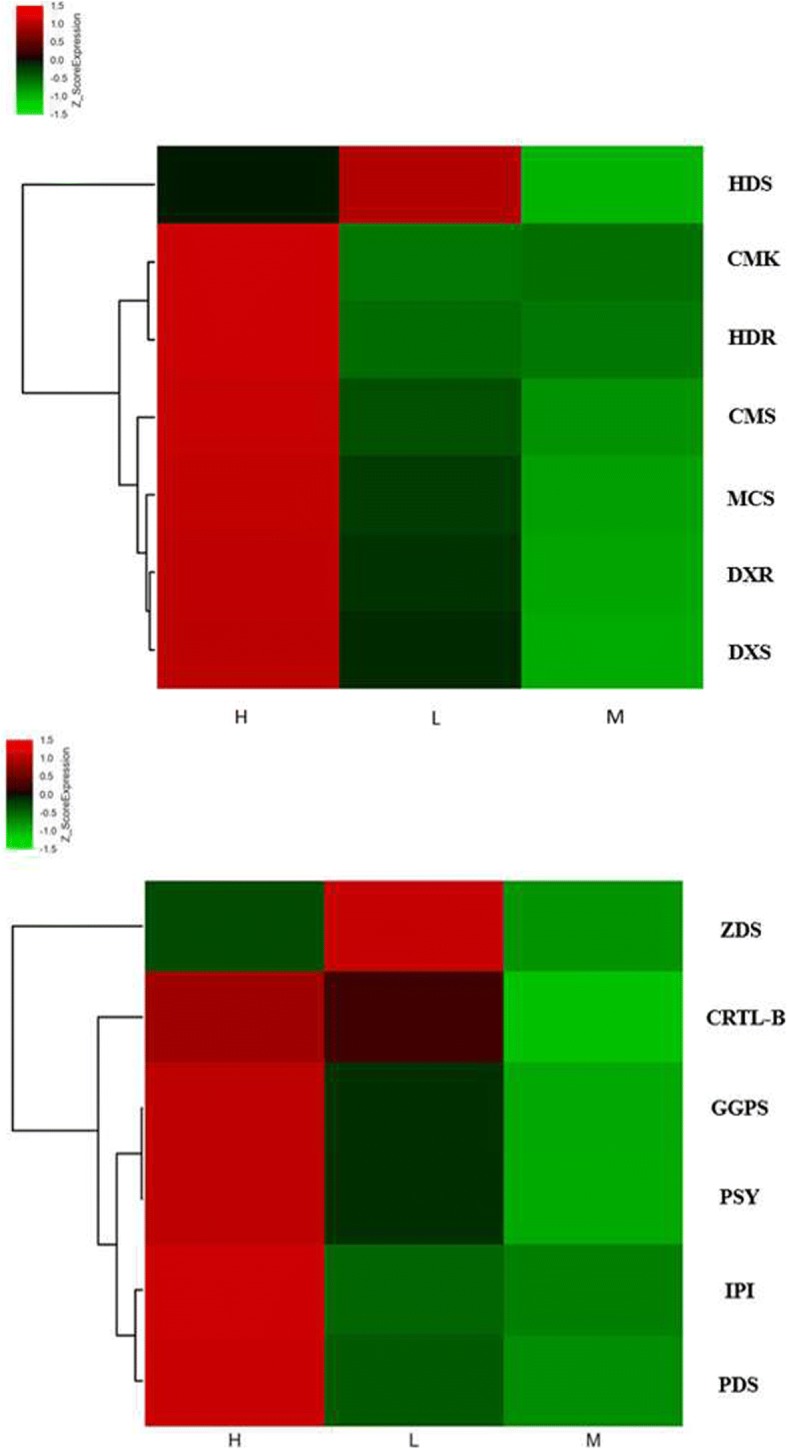
Heatmaps showing differentially expressed transcripts among L, M and H. Hierarchical clustering was performed with gene expression values using the program MeV. **a** Differentially expressed genes in the MEP pathway. The expression levels of HDR, CMS, CMK, MCS, DXS, and DXR were highest in H, and the expression level of HDS was highest in L. **b** Differentially expressed genes in the carotenoid biosynthetic pathway. The expression levels of CRTL-B, GGPS, PSY, IPI, and PDS were highest in H, and the expression level of ZDS was highest in L.

Heatmaps were constructed using the program MeV to show the differentially expressed transcripts among L, M and H (Fig. 8). Hierarchical clustering analysis was performed with seven genes of the MEP pathway for L, M and H (Fig. 8a). This analysis indicated two gene clusters. One cluster included the genes in L or H that were upregulated compared with the levels in M, namely, DXS, DXR, CMS, CMK, MCS and HDR; these genes had the highest expression levels in H. HDS grouped into the other cluster and had the highest expression level in L. In addition, hierarchical clustering analysis was performed with six genes of the carotenoid biosynthetic pathway (Fig. 8b). The results showed two gene clusters. One of the clusters, ZDS, this gene had the highest expression level in L. The other genes, GGPS, IPI, PSY, PDS, CRTL-B, grouped into the other cluster, and these genes had the highest expression level in H.

### qRT-PCR verification of changes in gene expression from the RNA-Seq analysis

**Fig. 9:**
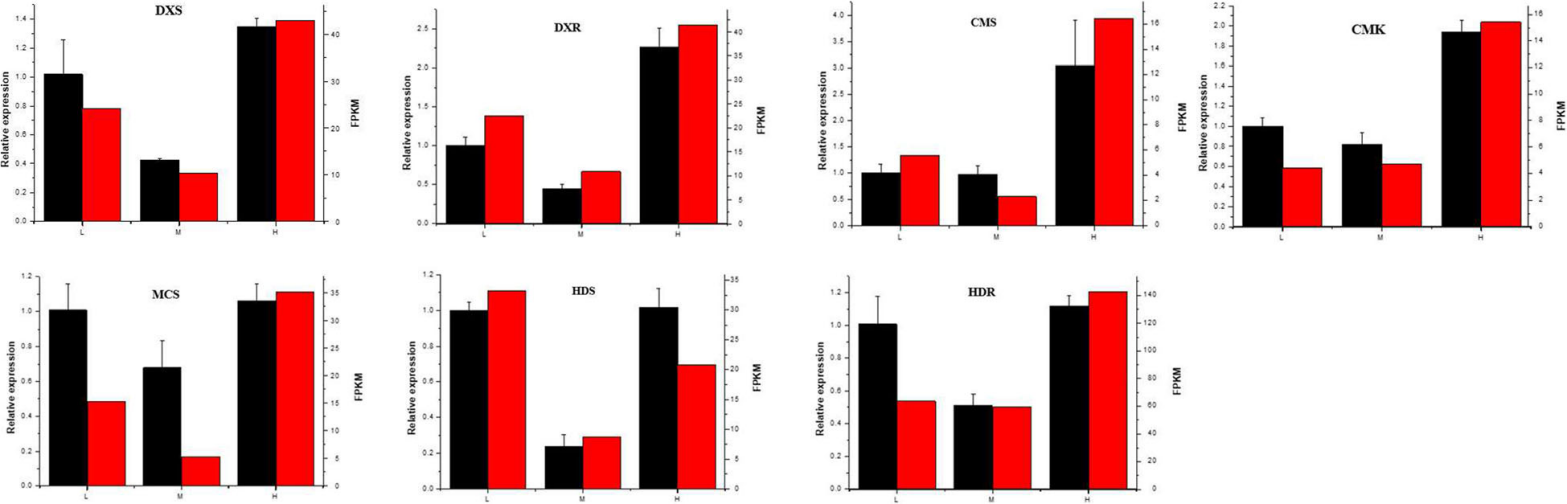
qRT-PCR verification of selected genes in the MEP pathway of *U. prolifera*. The black bars represent the relative expression determined with RT-qPCR (left y-axis), and the red bars represent the level of expression (FPKM) of the transcripts (right y-axis)

**Fig. 10:**
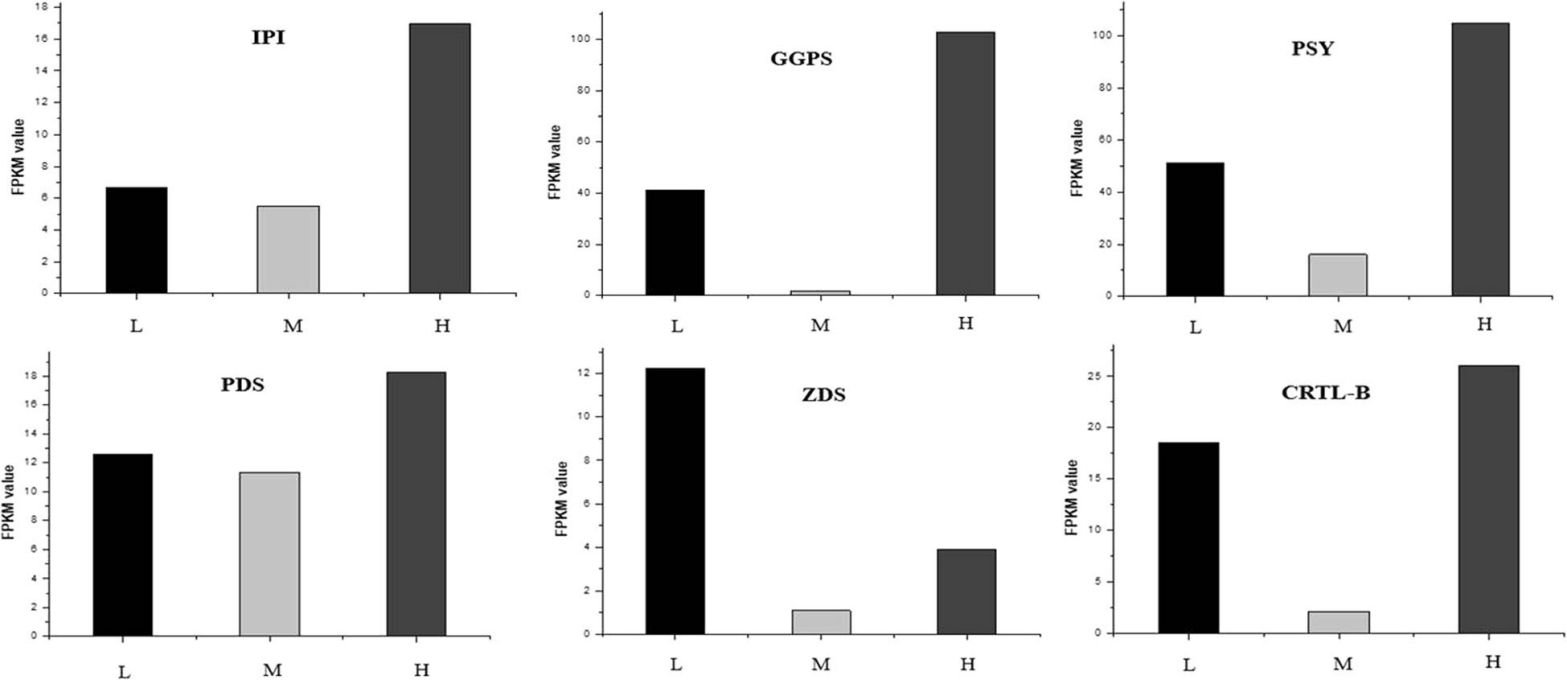
The FPKM values of GGPS, IPI, PSY, PDS, CRTL-B among L, M and H

To confirm the RNA-Seq results, the expression levels of all seven genes of the MEP pathway were quantified via qRT-PCR, and the expression levels (FPKM) among L, M and H were compared. There was a high degree of correlation between the qRT-PCR results and the RNA-Seq results (Fig. 9) indicating credibility of the transcriptomic profiling data. The FPKM values of GGPS, IPI, PSY, PDS, CRTL-B among L, M and H were compared simultaneously (Fig. 10). The expression levels of all the genes selected increased under low or high temperature, and our findings regarding the high expression of genes in the carotenoid biosynthetic pathway under low or high temperature were consistent with the high concentrations of carotenoids, Chl a and Chl b in the samples that were subjected to temperature stress.

## Discussion

Carotenoids have been studied for more than 100 years. They play important roles in the structure and function of the photosynthetic apparatus of living organisms, including bacteria, algae and higher plants [26]. More than 750 different carotenoids have been reported in nature [27]. The majority of carotenoids exist in the photosynthetic tissues of plants and algae, and the green colour of chlorophyll masks various carotenoids, carotenoids that are produced in photosynthetic tissues are not well known. Carotenoids are also widespread in photosynthetic bacteria and in microorganisms such as non-photosynthetic bacteria and yeast [28, 29]. Carotenoids have multiple functions, including enhancing immunity, inhibiting bacterial growth and exerting antioxidative activity [30, 31]. Many types of carotenoids have been identified from different plants, and they play an important role as antioxidants [32, 33]. Carotenoids participate in photoprotection in plants [34, 35]. In the past, carotenoid-related research has mostly focused on model species, such as maize, tomato, rice and Arabidopsis [36]. Many microalgae and macroalgae are rich in carotenoids, therefore, carotenoids extracted from algae may be the main natural resource for studying potential functional components [37]. However, genes for carotenogenesis in algae are not yet known [38]. There is also a lack of data on the expression patterns of genes related to carotenoid metabolism in *U. prolifera* and there have been limited attempts to understand carotenoid biosynthesis in this species. In this work, we analysed the carotenoid biosynthetic pathway in *U. prolifera* by transcriptome sequencing.

Functionally confirmed enzymes of carotenoid biosynthesis have been found in algae species such as *Chlorella*, *Chlamydomonas*, *Dunaliella* and *Haematococcus* [39–42]. Isopentenyl pyrophosphate (IPP), a C_5_-compound, is the source of chlorophylls and carotenoids. There are two pathways of synthesis of this precursor: the MVA pathway and the MEP pathway [43]. The pathway of carotenoid biosynthesis in algae is similar to that in plants and is dependent on the MVA or MEP pathway for precursor production. In green algae, some biochemical and genomic evidence has proven that the MVA pathway has been lost and that the MEP pathway is the sole pathway [44–47].

In our study, we found that some of the genes of the MVA pathway exist in *U. prolifera*, such as AACT and HMGR. The other genes, HMGS, MVK, PMK and MVD, were absent in the transcriptome data. This suggests that a complete MVA pathway to synthesize terpenoids is lacking in *U. prolifera*. The results were in accordance with the results of studies on *Porphyra umbilicalis*, *Cyanidioschyzon merolae* 10D, and *Chlorella zofingiensis* [48–50]. The MEP pathway exists in the plastid, which is the only source of terpenoids in green algae [51]. Some green algae regulate the flux of terpenoid metabolism by differential expression of the gene families of enzymes in the MEP pathway [52, 53].

Carotenoids are derived from the plastid-localized MEP pathway [54], for which pyruvate and glyceraldehyde 3-P act as initial substrates leading to the synthesis of GGPP [55]. Two GGPPs are catalysed by PSY to form phytoene [36]. Subsequently, carotenoids are produced from phytoene through a complex set of reactions requiring PDS, ZDS and CRTL-B [56]. We analysed the expression profiles of all the genes related to carotenoid biosynthesis and identified orthologues of previously known carotenoid genes in *U. perolifera*.

Secondary metabolites are the result of biological and non-biological interactions between organisms and the environment throughout evolution, and secondary metabolites play a critical role in improving the ability of organisms to survive and coordinate with the environment [25]. The production of and changes in secondary metabolites are influenced by the environment [57]. Plants have developed many modes for adaptation to temperature variations [58], and temperature is a main environmental factor that affects carotenoid biosynthesis and metabolism in plants [59–61] suggests different level of carotenoid synthesis and concentration according to change in temperature conditions. Both the carotenoid concentration and the expression of related genes were lowest under the normal temperature, while low temperature and high temperature seemed to activate the biosynthesis of carotenoids in *U. prolifera*. This finding revealed that carotenoids are involved in the response to temperature stress; in other words, carotenoids might have a protective function.

## Conclusion

In this study, we conducted transcriptomic and carotenoid biosynthetic analysis on samples of *U. prolifera* that were subjected to different temperatures. The results provided a comprehensive explanation of carotenoid biosynthesis in *U. prolifera*. The MEP pathway was detected from the transcriptome data. However, the MVA pathway was absent in terpenoid metabolism. Temperature is a key environmental factor affecting carotenoid biosynthesis. The production and concentrations of carotenoids were susceptible to temperature, and carotenoid concentrations were upregulated when *U. prolifera* were subjected to temperature stress. The data reported in this study provide critical information for understanding the accumulation of carotenoids and will serve as an important reference for the study of other metabolic pathways in *U. prolifera* that is the main species making green tide.

## Methods

### Plant materials

*U. prolifera* samples were collected in March 2018 from *Pyropia* rafts (32°26’N, 121°25′E) in Nantong, Jiangsu, China. The samples were cultivated in seawater medium, and cool-white fluorescent light was provided on a 12:12 L:D cycle. The cultivation environment of the samples was as follows: 120 μmol photons m^− 2^·s^− 1^ in seawater with a salinity of 30. There were three temperature regimes: 12 °C was set as the low temperature (L), 28 °C was set as the high temperature (H), and 20 °C was set as the medium temperature (M). The medium temperature group served as a control group. The samples, which were cultivated at the three different temperatures for 7 days, were frozen in liquid nitrogen until they were used for RNA extraction.

### Measurement of total carotenoids, chlorophyll (Chl) a and Chl b

Samples (0.05 g) from the three different temperatures were weighed, ground into powder, and placed into 5 mL of an 80% acetone solution. The samples were held at 4 °C for 12 h and then centrifuged at 10000 rpm for 20 min, and the supernatants were collected. The absorbances at wavelengths of 470 nm, 646.8 nm and 663.2 nm were measured using a spectrophotometer (Hitachi, Japan), and the quantitative determination of carotenoid, Chl a and Chl b levels was performed with the following formulas [62]:

Total carotenoids (μg/ml) = (1000 × A_470_ – 1.82 × Chl a – 85.02 × Chl b)/198

Chl a (μg/ml) = 12.25 × A_663.2_ – 2.79 × A_648.8_

Chl b (μg/ml) = 21.5 × A_646.8_ – 5.1 × A_663.2_

### RNA extraction

Total RNA from all samples was extracted using an E.Z.N.A.® Plant RNA Kit Omega Bio-tek, USA), and an aliquot of total RNA was treated with DNase I (Takara, China) to remove DNA. RNA integrity was measured by 1% agarose gel electrophoresis, and RNA purity was detected with a NanoDrop spectrophotometer (Thermo Fisher, USA).

### Illumina HiSeq library preparation and sequencing

These RNA samples were reverse transcribed into cDNA using a SMARTer™ PCR cDNA Synthesis Kit (Takara, China), and cDNA libraries were created. After the quality of the cDNA libraries was assessed with an Agilent Bioanalyzer 2100 system (Agilent Technologies, USA), sequencing was conducted using an Illumina HiSeq X Ten sequencer by Shanghai OE Biotech. Co., Ltd. The raw data were uploaded to the NCBI Sequence Read Archive (SRA, https://trace.ncbi.nlm.nih.gov/Traces/sra/sra.cgi). The data and scripts used for the analysis is available with accession number SRP157932.

### Data analysis and annotation

Raw reads were quality filtered using the Trimmomatic 0.36 [63], and the process consisted of four stages: removal of the adaptor; removal of low-quality reads for which the number of N bases exceeded 10% of the total read length or for which the number of error-prone bases (quality score ≤ 5) exceeded 50% of the total read length; removal of low-quality bases from the 3′ end and the 5′ end in different ways; and statistical analysis of the raw reads and clean reads.

The data were assessed for contamination before subsequent analysis; 250,000 pairs of reads (500,000 reads) from the data were extracted randomly, and then the data were aligned using BLAST (E value < 10^− 10^, coverage > 80%) with sequences in the National Center for Biotechnology Information (NCBI) non-redundant nucleotide sequence (Nt) database (ftp://ftp.ncbi.nih.gov/blast/db). The best dataset was selected.

Gene function was annotated using the DIAMOND 4.0 program [64] with an E-value cut-off of 1e− 5 against the following databases: NCBI non-redundant protein sequences (Nr, https://blast.ncbi.nlm.nih.gov/), the EuKaryotic Orthologous Groups (KOG) database (http://www.ncbi.nlm.nih.gov/COG/), the Gene Ontology (GO) database (http://www.geneontology.org), the Swiss-Prot database (http://www.uniprot.org/), the evolutionary genealogy of genes: Non-supervised Orthologous Groups (eggNOG) (http://eggnogdb.embl.de/) database and the Kyoto Encyclopedia of Genes and Genomes (KEGG) database (http://www.genome.jp/kegg/pathway.html). We screened for proteins with the highest sequence similarity for functional annotation information. In addition, the HMMER program [65] was used against the protein families (Pfam) database (http://pfam.xfam.org/) to screen the protein family with the highest score.

### Differential expression analysis

Analysis of the differential expression of unigenes among different samples was conducted using DESeq [66], a method based on the negative binomial distribution, in the R statistical environment [67]. The number of unigenes in each sample was normalized using the baseMean value to estimate the expression, the fold change was calculated, and the significance of the difference in the number of reads was tested with an NB test (negative binomial distribution test). Finally, we screened for the differential expression of unigenes based on the fold changes and the results of the significance test.

### Phylogenetic analysis of carotenoid biosynthesis genes

The expression profiles of genes involved in terpenoid biosynthesis were analysed using KEGG pathway annotation. A total of 56 expressed unigenes encoding terpenoid biosynthesis enzymes were found in *U. prolifera*. But most of the genes encoding key enzymes in the MVA pathway were not found, except for AACT and HMGR. In addition, a total of 22 expressed unigenes encoding carotenoid biosynthesis enzymes were found, and six genes encoding key enzymes in carotenoid biosynthesis were identified in *U. prolifera*. Functional genes that participate in terpenoid biosynthesis in *Chlamydomonas reinhardtii* and *Chlorella variabilis* were selected from NCBI and aligned with the sequences of *U. prolifera*. The phylogenetic analysis was carried out in MEGA 5.1 software [68] with the neighbour-joining (NJ) analysis option based on the amino acid sequences.

### Expressional validation of carotenoid biosynthesis genes with qRT-PCR

The expression levels of the seven unigenes that participate in the MEP pathway were examined by qRT-PCR. The primers for qRT-PCR are shown in Table 2. The expression levels were measured with a Baiyuan ASA-4800 Real Time PCR System using SYBR Green fluorescent dye (Takara, China) according to the manufacturer’s instructions. The cycling profile included a step at 95 °C for 30 s, followed by 40 cycles of amplification (95 °C for 5 s and 60 °C for 34 s). The relative gene expression was calculated using the 2^-ΔΔCt^ relative quantitative method. All MEP pathway genes were chosen for analysis of expression patterns in different samples that were subjected to the three different temperatures (12 °C, 20 °C, and 28 °C). The expression levels of these selected genes from qRT-PCR analyses were assessed in comparison to the differentially expressed genes (DEGs) from RNA-Seq.

**Table 2:** Design primers for Real-Time PCR

Gene name	→Forward primer (5′ 3′)	→Reverse primer (5′ 3′)
18S rDNA	CGCAACTCCCGACTCACGAAGG	ATCTTTGAGACAAGCATATGACTAC
DXS	GCCTGTGCGGTTTGCGATGGAT	ACTTCCAGCAACACGGCATCGG
DXR	CGGGGAAGATGTTGATGCGCTGAA	AAAACACGGCGTCCACATCCTTCC
CMS	GGTTCTACTCGCTGGTGGCGTTGG	CTTGGCGCTCCGCTCCTGGTAA
CMK	TACAGCGTTGCCAAGCGTACCAAA	CATGGAAAAGCGACGCCAAATCA
MCS	CTTGTTGTTCGGTGATGTGGCTCG	CCGGTTTGGCTTGTTTGAAGGTCC
HDS	CTGGGAACTTTGCAGACGGAACGA	AAGACAGAATGCGGGCACTCAAGC
HDR	TTGCGTGGGAGGGGCGTGTTAC	AGGTCAGGTTGGTGGCAACATGGG

## Additional files

Additional file 1: **Figure S1.** GO annotation of the non-redundant sequences of all the samples. Three primary GO categories and 56 subcategories were summarized in the GO database. (TIF 9821 kb)

Additional file 2: **Figure S2.** GO terms significantly enriched in DEGs in comparisons of L, M and H. (TIF 27551 kb)

## Abbreviations

bp: Base pairs
CMK: 4-(cytidine-5-diphospho)-2-C-methyl-D-erythritol kinase
CMS: 2-C-methyl-D-erythritol 4-phosphate cytidyltransferase
CRTL-B: lycopene b-cyclase
DEGs: Differentially expressed genes
DXR: 1-deoxy-D-xylulose 5-phosphate reductoisomerase
DXS: 1-deoxy-D-xylulose 5-phosphate synthase
FPKM: Fragments per kilobase of exon per million fragments mapped
GGPS: Geranylgeranyl diphosphate synthase
GO: Gene ontology
HDR: 1-hydroxy-2-methyl-2-(*E*)-butenyl-4-diphosphate reductase
HDS: 1-hydroxy-2-methyl-2-(*E*)-butenyl-4-diphosphate synthase
IPI: Isopentenyl diphosphate isomerase
KEGG: Kyoto encyclopedia of genes and genomes
KOG: Eukaryotic Orthologous Groups
MCS: 2-C-methyl-D-erythritol 2,4-cyclodiphosphate synthase
MEP: 2-C-methyl-D-erythritol 4-phosphate
MVA: Mevalonate acid
Nr: NCBI non-redundant protein sequences
PDS: Phytoene desaturase
PSY: Phytoene synthase
qRT-PCR: Quantitative reverse transcription PCR
RNA-Seq: RNA sequencing
ZDS: ζ-carotene desaturase

## References

[1] Lange BM, Rujan T, Martin W, Croteau R (2000). Isoprenoid biosynthesis: the evolution of two ancient and distinct pathways across genomes. Proc Natl Acad Sci U S A.

[2] Sharma E, Anand G, Kapoor R (2017). Terpenoids in plant and arbuscular mycorrhiza-reinforced defence against herbivorous insects. Ann Bot.

[3] Lohr M, Schwender J, Jew P (2012). Isoprenoid biosynthesis in eukaryotic phototrophs: a spotlight on algae. Plant Sci.

[4] Vranová E (2012). Systems Understanding of isoprenoid pathway regulation in Arabidopsis.

[5] Tetraterpenes TS (2013). Carotenoids.

[6] Sun G, Sui Z, Zhang X (2008). Cloning and characterization of the phytoene desaturase(pds) gene-a key enzyme for carotenoids synthesis in *Dunaliella* (Chlorophyta). J Ocean Univ China.

[7] Chen F, Tholl D, Bohlmann J, Pichersky E (2011). The family of terpene synthases in plants: a mid-size family of genes for specialized metabolism that is highly diversified throughout the kingdom. Plant J.

[8] Jia Q. Analysis of OneKP transcriptomes reveals unequal distribution of terpene synthase genes across diverse taxa in the plant kingdom. Plant Animal Genome. 2015. p 1009.

[9] Wu C, Peng J, Yang G, Liu J, Jin Z, Fu H (2017). Isolation and characterization of *Ulva prolifera* actin1 gene and function verification of the 5′ flanking region as a strong promoter. Bioengineered.

[10] Hiraoka M, Dan A, Shimada S, Hagihira M, Migita M, Ohno M (2003). Different life histories of *Enteromorpha prolifera* (Ulvales, Chlorophyta) from four rivers on Shikoku Island, Japan. Phycologia.

[11] Duan W, Guo L, Sun D, Zhu S, Chen X, Zhu W (2012). Morphological and molecular characterization of free-floating and attached green macroalgae *Ulva spp*. in the Yellow Sea of China. J Appl Phycol.

[12] Liu D, Keesing JK, Dong Z, Zhen Y, Di B, Shi Y (2010). Recurrence of the world's largest green-tide in 2009 in Yellow Sea, China: *Porphyra yezoensis* aquaculture rafts confirmed as nursery for macroalgal blooms. Mar Pollut Bull.

[13] Fan S, Mingzhu FU, Yan LI, Wang Z, Fang S, Jiang M (2012). Origin and development of Huanghai (yellow) sea green-tides in 2009 and 2010. Acta Oceanol Sin.

[14] Zhang J, Huo Y, Wu H, Yu K, Kim JK, Yarish C (2014). The origin of the *Ulva* macroalgal blooms in the Yellow Sea in 2013. Mar Pollut Bull.

[15] Liu Q, Yu RC, Yan T, Zhang QC, Zhou MJ (2015). Laboratory study on the life history of bloom-forming *Ulva prolifera* in the Yellow Sea. Estuar Coast Shelf Sci.

[16] Huang XY, Zang XN, Wu F, Jin YM, Wang HT, Liu C (2017). Transcriptome sequencing of Gracilariopsis lemaneiformis to analyze the genes related to optically active Phycoerythrin synthesis. PLoS One.

[17] Chen J, Tang X, Ren C, Wei B, Wu Y, Wu Q (2018). Full-length transcriptome sequences and the identification of putative genes for flavonoid biosynthesis in safflower. BMC Genomics.

[18] Chen C, Zheng Y, Zhong Y, Wu Y, Li Z, Xu LA (2018). Transcriptome analysis and identification of genes related to terpenoid biosynthesis in Cinnamomum camphora. BMC Genomics.

[19] Carniel FC, Gerdol M, Montagner A, Banchi E, Moro GD, Manfrin C (2016). New features of desiccation tolerance in the lichen photobiont Trebouxia gelatinosa are revealed by a transcriptomic approach. Plant Mol Biol.

[20] Kumaresan V, Nizam F, Ravichandran G, Viswanathan K, Palanisamy R, Bhatt P (2017). Transcriptome changes of blue-green algae, Arthrospira sp in response to sulfate stress. Algal Res.

[21] Sun P, Mao Y, Li G, Cao M, Kong F, Wang L (2015). Comparative transcriptome profiling of *Pyropia yezoensis* (Ueda) M.S. Hwang & H.G. Choi in response to temperature stresses. BMC Genomics.

[22] Fan J, Xu H, Li Y (2016). Transcriptome-based global analysis of gene expression in response to carbon dioxide deprivation in the green algae *Chlorella pyrenoidosa*. Algal Res.

[23] He BX, Hou LL, Dong MM, Shi JW, Huang XY, Ding YT (2018). Transcriptome analysis in *Haematococcus pluvialis*: Astaxanthin induction by high light with acetate and Fe^2+^. Int J Mol Sci.

[24] Puente-Sanchez F, Diaz S, Penacho V, Aguilera A, Olsson S (2018). Basis of genetic adaptation to heavy metal stress in the acidophilic green alga *Chlamydomonas acidophila*. Aquat Toxicol.

[25] He Y, Yan Z, Du Y, Ma Y, Shen S (2017). Molecular cloning and expression analysis of two key genes, HDS and HDR, in the MEP pathway in *Pyropia haitanensis*. Sci Rep.

[26] Tran D, Haven J, Qiu WG, Polle JE (2009). An update on carotenoid biosynthesis in algae: phylogenetic evidence for the existence of two classes of phytoene synthase. Planta.

[27] Nisar N, Li L, Lu S, Khin NC, Pogson BJ (2015). Carotenoid metabolism in plants. Mol Plant.

[28] Frengova G, Simova E, Pavlova K, Beshkova D, Grigorova D (1994). Formation of carotenoids by rhodotorula glutinis in whey ultrafiltrate. Biotechnol Bioeng.

[29] Frengova G, Simova E, Beshkova D (2004). Use of whey ultrafiltrate as a substrate for production of carotenoids by the yeast Rhodotorula rubra. Appl Biochem Biotechnol.

[30] Giovannucci E (1999). Tomatoes, tomato-based products, lycopene, and cancer: review of the EpidemiologicLiterature. J Natl Cancer Inst.

[31] Agarwal S, Rao AV (2000). Tomato lycopene and its role in human health and chronic diseases. Can Med Assoc J.

[32] Qin G, Gu H, Ma L, Peng Y, Deng XW, Chen Z, et al. Disruption of phytoene desaturase gene results in albino and dwarf phenotypes in Arabidopsis by impairing chlorophyll, carotenoid, and gibberellin biosynthesis. Cell Res. 2007;17(5):471–82. 10.1038/cr.2007.40 17486124

[33] Avendaño-Vázquez AO, Cordoba E, Llamas E, San RC, Nisar N, De lTS (2014). An uncharacterized apocarotenoid-derived signal generated in ζ-carotene desaturase mutants regulates leaf development and the expression of chloroplast and nuclear genes in arabidopsis. Plant Cell.

[34] Kim JY, Smith JJ, Tian L, Dellapenna D (2009). The evolution and function of carotenoid hydroxylases in *Arabidopsis*. Plant Cell Physiol.

[35] Cazzaniga S, Li Z, Niyogi KK, Bassi R, Dall'Osto L (2012). The Arabidopsis szl1 mutant reveals a critical role of β-carotene in photosystem I photoprotection. Plant Physiol.

[36] Cazzonelli CI, Pogson BJ (2010). Source to sink: regulation of carotenoid biosynthesis in plants. Trends Plant Sci.

[37] Christaki E, Bonos E, Giannenas I, Paneri PF (2012). Functional properties of carotenoids originating from algae. J Sci Food Agric.

[38] Shinichi T (2011). Carotenoids in algae: distributions, biosyntheses and functions. Mar Drugs.

[39] Huang J, Liu J, Li Y, Chen F (2010). Isolation and characterization of the phytoene desaturase gene as a potential selective marker for genetic engineering of the astaxanthin-producing green alga *chlorella zofingiensis* (Chlorophyta). J Phycol.

[40] Mccarthy SS, Kobayashi MC, Niyogi KK (2004). White mutants of *Chlamydomonas reinhardtii* are defective in phytoene synthase. Genetics.

[41] Ramos A, Coesel S, Marques A, Rodrigues M, Baumgartner A, Noronha J (2008). Isolation and characterization of a stress-inducible *Dunaliella salina* Lcy-beta gene encoding a functional lycopene beta-cyclase. Appl Microbiol Biotechnol.

[42] Steinbrenner J, Linden H (2001). Regulation of two carotenoid biosynthesis genes coding for phytoene synthase and carotenoid hydroxylase during stress-induced astaxanthin formation in the green alga *Haematococcus pluvialis*. Plant Physiol.

[43] Eisenreich W, Bacher A, Arigoni D, Rohdich F (2004). Biosynthesis of isoprenoids via the non-mevalonate pathway. Cell Mol Life Sci.

[44] Disch A, Schwender J, Muller C, Lichtenthaler H, Rohmer M (1998). Distribution of the mevalonate and glyceraldehyde phosphate/pyruvate pathways for isoprenoid biosynthesis in unicellular algae and the cyanobacterium Synechocystis PCC 6714. Biochem J.

[45] Schwender J, Seemann M, Lichtenthaler HK, Rohmer M (1996). Biosynthesis of isoprenoids (carotenoids, sterols, prenyl side-chains of chlorophylls and plastoquinone) via a novel pyruvate/glyceraldehyde 3-phosphate non-mevalonate pathway in the green alga *Scenedesmus obliquus*. Biochem J.

[46] Grauvogel C, Petersen J (2007). Isoprenoid biosynthesis authenticates the classification of the green alga *Mesostigma viride* as an ancient streptophyte. Gene.

[47] Grossman AR, Lohr M, Im CS (2004). *Chlamydomonas reinhardtii* in the landscape of pigments. Annu Rev Genet.

[48] Chan CX, Blouin NA, Zhuang Y, Zäuner S, Prochnik SE, Lindquist E (2012). *Porphyra* (Bangiophyceae) transcriptomes provide insights into red algal development and metabolism. J Phycol.

[49] Matsuzaki M, Misumi O, Shini T, Maruyama S, Takahara M, Miyagishima S (2004). Genome sequence of the ultrasmall unicellular red alga *Cyanidioschyzon merolae* 10D. Nature.

[50] Huang W, Ye J, Zhang J, Lin Y, He M, Huang J (2016). Transcriptome analysis of *Chlorella zofingiensis* to identify genes and their expressions involved in astaxanthin and triacylglycerol biosynthesis. Algal Res.

[51] Schwender J, Lichtenthaler HK (2001). Chlorophyta exclusively use the 1-deoxyxylulose5-phosphate/2-C-methylerythritol 4-phosphate pathway for the biosynthesisof isoprenoids. Planta.

[52] Jin E, Lee CG, Polle JEW (2006). Secondary carotenoid accumulation in *Haematococcus* (Chlorophyceae): biosynthesis, regulation, and biotechnology. J Microbiol Biotechnol.

[53] Jin ES, Polle JR (2009). Carotenoid biosynthesis in *Dunaliella* (Chlorophyta).

[54] Phillips MA, León P, Boronat A, Rodríguez-Concepción M (2008). The plastidial MEP pathway: unified nomenclature and resources. Trends Plant Sci.

[55] Ganjewala D, Kumar S, Luthra R (2009). An account of cloned genes of methyl-erythritol-4-phosphate pathway of isoprenoid biosynthesis in plants. Curr Issues Mol Biol.

[56] Li F, Murillo C, Wurtzel ET (2007). Maize Y9 encodes a product essential for 15-cis-ζ-carotene isomerization. Plant Physiol.

[57] Kliebenstein DJ (2010). Secondary metabolites and plant/environment interactions: a view through Arabidopsis thaliana tinged glasses. Plant cell. Environment.

[58] Kopsell DA, Lefsrud MG, Kopsell DE, Curran-Celentano J (2005). Air temperature affects biomass and carotenoid pigment accumulation in kale and spinach grown in a controlled environment. Hortsci Publ Am Soc Horticult Sci.

[59] Haldimann P (2010). Effects of changes in growth temperature on photosynthesis and carotenoid composition in zea mays leaves. Physiol Plant.

[60] Matsumoto H, Ikoma Y, Kato M, Nakajima N, Hasegawa Y (2009). Effect of postharvest temperature and ethylene on carotenoid accumulation in the Flavedo and juice sacs of Satsuma mandarin (Citrus unshiu Marc.) fruit. J Agric Food Chem.

[61] Ezell BD, Wilcox MS (1952). Influence of storage temperature on carotene, Total carotenoids and Asorbic acid content of Sweetpotatoes. Plant Physiol.

[62] Lichtenthaler HK, Buschmann C. Chlorophylls and Carotenoids: Measurement and characterization by UV-VIS spectroscopy. John Wiley & Sons, Inc. Current Protocols in Food Analytical Chemistry (CPFA). 2001;39(6):1230–7.

[63] Bolger AM, Lohse M, Usadel B (2014). Trimmomatic:a flexible trimmer for Illumina sequence data. Bioinformatics.

[64] Buchfink B, Xie C, Huson DH (2015). Fast and sensitive protein alignment using DIAMOND. Nat Methods.

[65] Mistry J, Finn RD, Eddy SR, Bateman A, Punta M (2013). Challenges in homology search: HMMER3 and convergent evolution of coiled-coil regions. Nucleic Acids Res.

[66] Anders S, Huber W. Differential expression of RNA-Seq data at the gene level – the DESeq package. Embl 2012.

[67] Team RDC (2010). R: a language and environment for statistical computing.

[68] Hall BG (2013). Building phylogenetic trees from molecular data with MEGA. Mol Biol Evol.

