# Supplementary figures and images for: Differential gene expression for carotenoid biosynthesis in a green alga Ulva prolifera based on transcriptome analysis
Source: BMC Genomics. 2018 Dec 13;19:916. doi: 10.1186/s12864-018-5337-y (PMC6293516; doi:10.1186/s12864-018-5337-y)

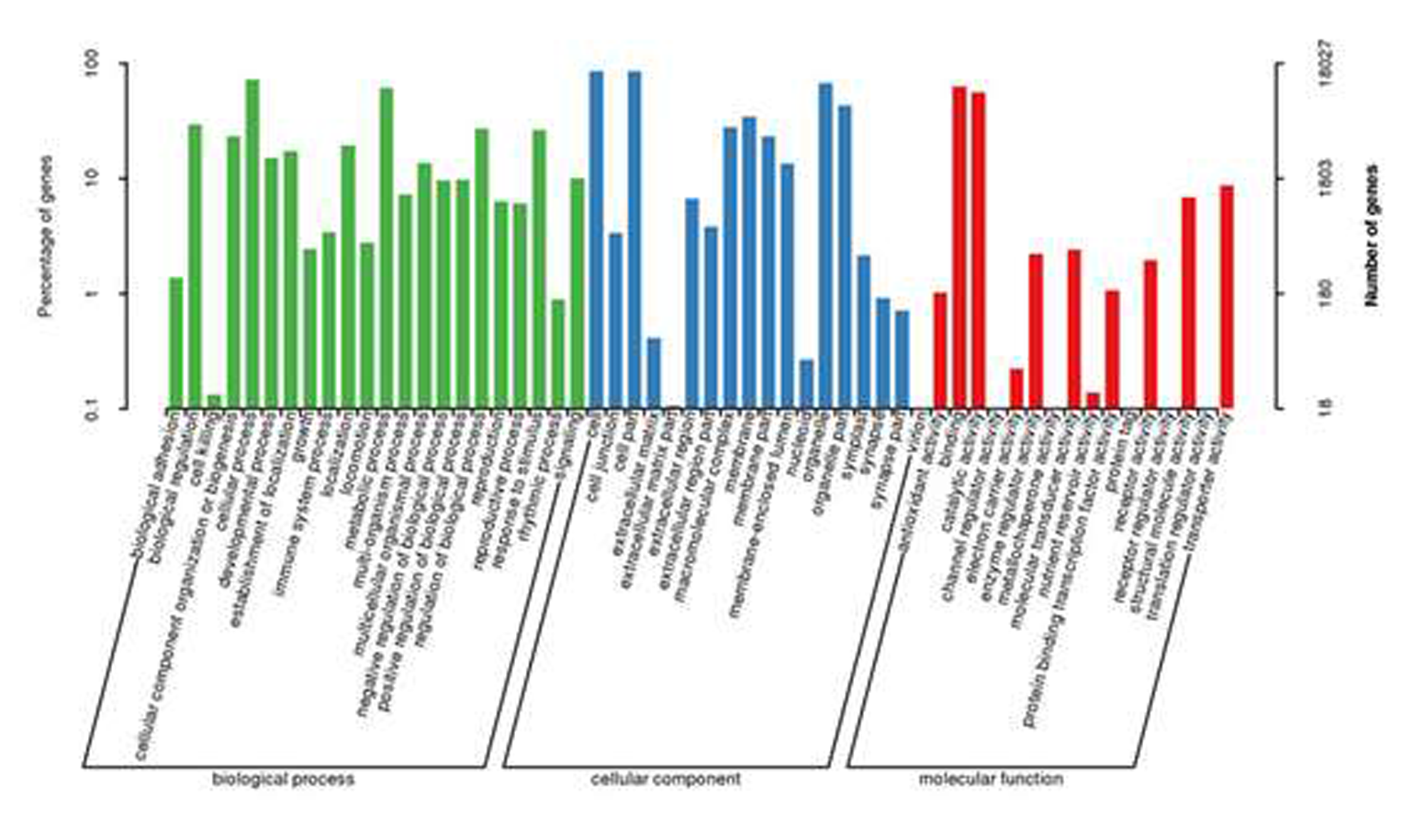

Supplement: Supplementary file 1 — Figure S1. GO annotation of the non-redundant sequences of all the samples. Three primary GO categories and 56 subcategories were summarized in the GO database. (TIF 9821 kb) [file 12864_2018_5337_MOESM1_ESM.tif]

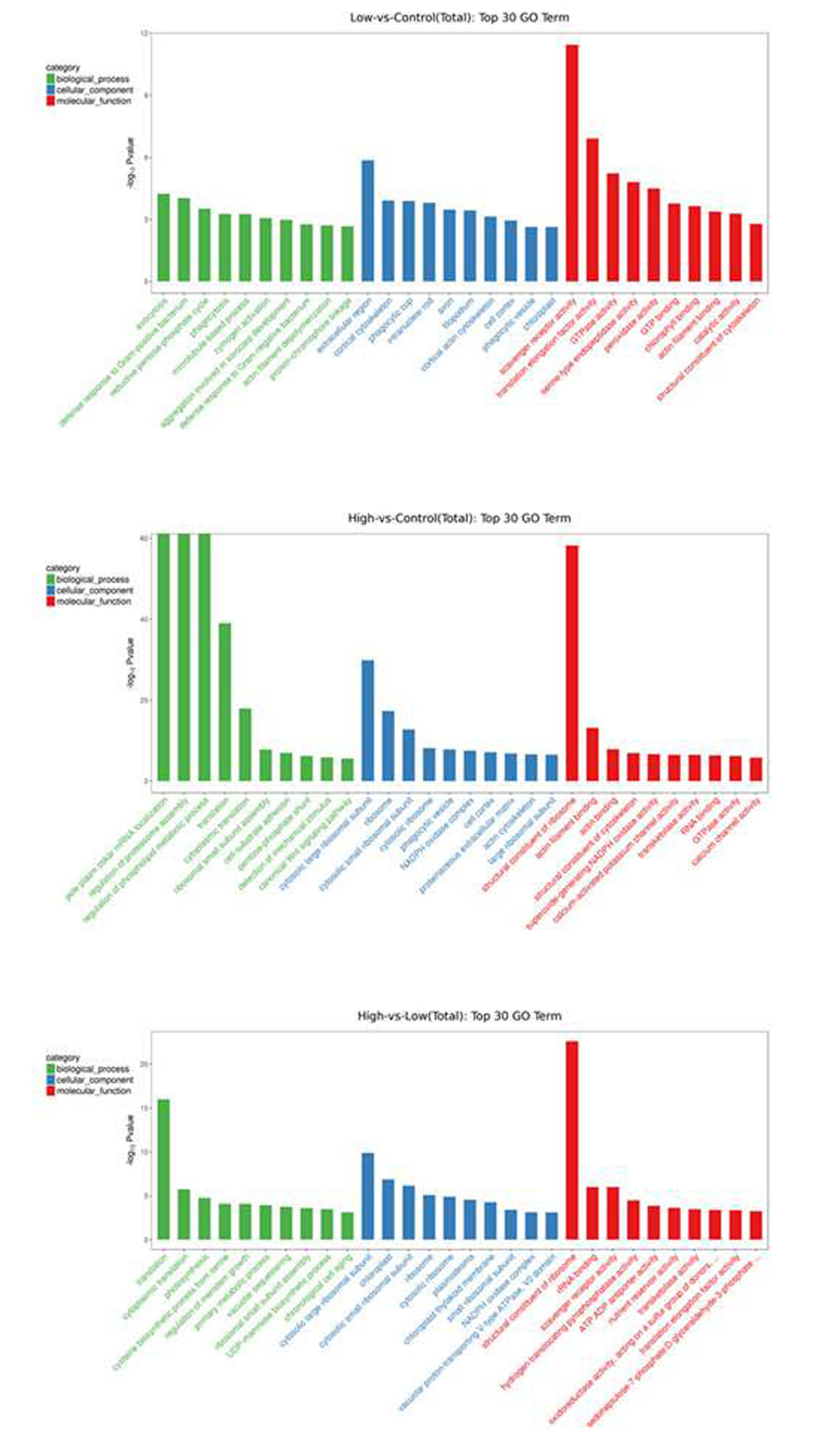

Supplement: Supplementary file 2 — Figure S2. GO terms significantly enriched in DEGs in comparisons of L, M and H. (TIF 27551 kb) [file 12864_2018_5337_MOESM2_ESM.tif]
